# Return on investments in the Health Extension Program in Ethiopia

**DOI:** 10.1371/journal.pone.0291958

**Published:** 2023-11-27

**Authors:** Diana Bowser, Eckhard Kleinau, Grace Berchtold, David Kapaon, Leulsegged Kasa

**Affiliations:** 1 Heller School for Social Policy and Management, Brandeis University, Waltham, MA, United States of America; 2 University Research Co. Chevy Chase, Chevy Chase, MD, United States of America; 3 Harvard Center for Population and Development Studies, Harvard T.H. Chan School of Public Health, Cambridge, MA, United States of America; Jimma University, ETHIOPIA

## Abstract

**Background:**

Since 2003, the government of Ethiopia has trained and deployed more than 42,000 Health Extension Workers across the country to provide primary healthcare services. However, no research has assessed the return on investments into human resources for health in this setting. This study aims to fill this gap by analyzing the return on investment within the context of the Ethiopian Health Extension Program.

**Methods:**

We collected data on associated costs and benefits attributed to the Health Extension Program from primary and secondary sources. Primary sources included patient exit interviews, surveys with Health Extension Workers and other health professionals, key informant interviews, and focus groups conducted in the following regions: Amhara, Oromia, Tigray, and the Southern Nations Nationalities and Peoples’ Region. Secondary sources consisted of financial and administrative reports gathered from the Ministry of Health and its subsidiaries, as well as data accessed through the Lives Saved Tool. A long-run return on investment analysis was conducted considering program costs (personnel, recurrent, and capital investments) in comparison to benefits gained through improved productivity, equity, empowerment, and employment.

**Findings:**

Between 2008–2017, Health Extension Workers saved 50,700 maternal and child lives. Much of the benefits were accrued by low income, less educated, and rural women who had limited access to services at higher level health centers and hospitals. Regional return ranged from $1.27 to $6.64, with an overall return on investment in the range of $1.59 to $3.71.

**Conclusion:**

While evidence of return on investments are limited, results from the Health Extension Program in Ethiopia show promise for similar large, sustainable system redesigns. However, this evidence needs to be contextualized and adapted in different settings to inform policy and practice. The Ethiopian Health Extension Program can serve as a model for other nations of a large-scale human resources for health program containing strong economic benefits and long-term sustainability through successful government integration.

## Introduction

Developing countries have shown increasing interest in utilizing community health workers (CHWs) to improve health outcomes and strengthen health systems. Such interests stem from the CHW’s effectiveness in providing promotive, preventive, and curative health services at the community level [1, 2]. As a result, CHWs have become the second most common type of health workers in Africa only surpassed by nursing staff [3]. In particular, Ethiopia has been an exemplary country in further developing the scope of practice for CHWs, creating a new cadre of health workers called Health Extension Workers (HEWs), who act as professional community nurses and are the primary inputs to the Health Extension Program (HEP). Operational since 2003, the HEP seeks to deliver primary health services at the community and household level through HEWs [4].

Ethiopia has significantly invested in HEWs in order to increase access to primary health care in rural areas. More than 80 percent of the country’s population lives outside of urban locales where access to health services is very limited [5–7]. As a result, the government of Ethiopia began training and deploying salaried HEWs to provide care in these underserved areas [5]. Since the inception of the HEP back in 2003, more than 42,000 HEWs have been deployed to provide primary level health promotion, prevention, and curative services to households and communities across the country [5]. HEWs have been a foundational aspect for making the HEP’s larger goals become a reality [4, 8–10].

HEWs have played a key role in improving access to primary health care in Ethiopia. For instance, past research has found that HEWs increase child immunization coverage, potential health service coverage, as well as outpatient attendance per capita in the country [11]. In addition, one study reported significant associations between HEWs and maternal and child health services utilization [12]. Furthermore, additional research found HEWs were partially responsible for antenatal care coverage increasing from 26.8% to 42.5% between 2000–2011 [13]. Such success stories pushed the Ethiopian government to expand the HEP and begin training and deploying HEWs into pastoral and urban areas as well [4, 8, 14]. While recent research identified the mechanisms through which HEWs contribute to higher performing administrative districts [2], no study to date has assessed the return of such investments into human resources for health, incorporating equity and empowerment benefits.

Return on investment (ROI) calculations are important analyses that compare the value of an investment against any associated costs [15–21]. It is an economic approach computed as the ratio of net benefits (benefits minus costs) to costs in terms of monetary value [16]. ROI analyses have been used extensively in high-income settings to understand the impact of reductions in health care spending through “cost offsets”, such as emergency department use, readmission rates, and hospital inpatient visits. ROIs are becoming frequently used in low-income settings to measure the impact of various health care programs, including community health worker programs [20].

## Methods

### Study setting

This study used a simultaneous mixed methods approach, relying on quantitative and qualitative data collected from the following four regions in Ethiopia: Amhara, Oromia, Tigray, and the Southern Nations, Nationalities and People’s region (SNNPR). Eighty-seven percent of the country’s population lives in these four regions, and the HEP has been operational in Tigray since its inception in 2003. For this study, a single district per region was randomly selected for data collection as follows: Angolella Tera in Amhara, Awasa Zuria in SNNPR, Kilite Awulaelo in Tigray, and Tiyo in Oromia. Within each district, four health centers (HCs) and 15 health posts (HPs) were utilized as the primary and secondary data sources.

### Return on investment framework

**Fig 1:**
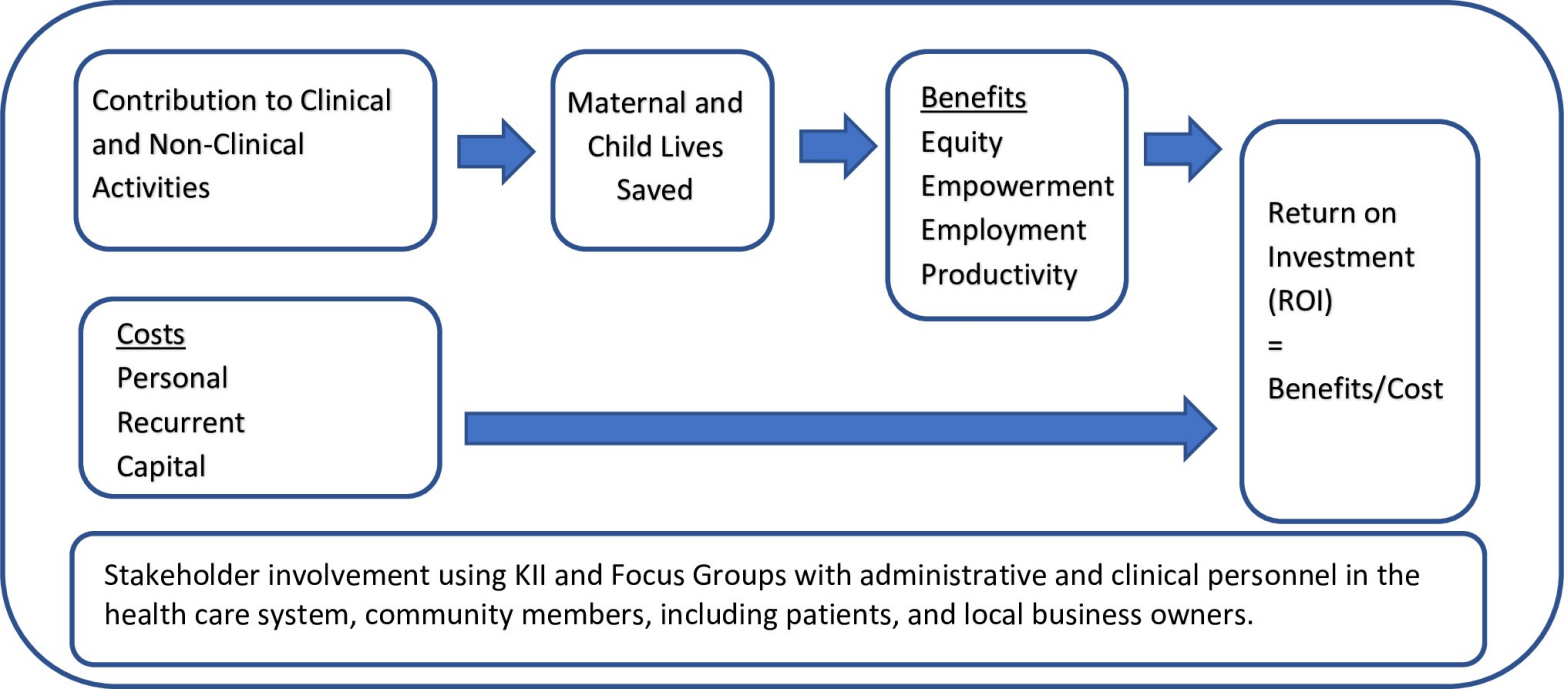
Return on investment framework of the Health Extension Program in Ethiopia. Fig 1 captures the theory of change and framework developed by the research team and key stakeholders to understand how the additional clinical and non-clinical services provided through the Health Extension Program should impact the lives of woman and children resulting in key benefits captured through equity, empowerment, employment, and productivity. Total benefits are compared with the total costs needed to implement the program to calculate the return on investment (ROI).

The ROI analysis of CHWs can be conceptualized through the framework presented in Fig 1. The top row captures how the research team and stakeholders used theory of change to understand how the HEP impacted the health system. By investing in human resources for health, the HEP contributed to improving clinical and non-clinical activities with a focus on women and their children. As a result, the framework hypothesizes that maternal and child lives would not only be saved, but the following benefits would be accrued as well: increased equity, empowerment, employment, and productivity. The main inputs were the additional human resources that were added to the Ethiopian health care system as part of the HEP. These benefits were compared against program costs, measured as investments in personnel (human resources), recurrent costs, and capital items.

### Data sources

We collected data on associated costs and benefits attributed to the HEP from primary and secondary sources. Primary data sources included patient exit interviews at both HCs and HPs, as well as surveys with HEWs and interviews with other health professionals at HCs. In addition, we conducted key informant interviews (KII) with administrative personnel within the Ethiopian health care system at the zonal, regional, and national levels to gather cost, administrative, and implementation data. Additional focus group discussions (FGD) were conducted with community members and local business owners to gather data about perceptions of the HEP, and local spending patterns, respectively. Informed consent was collected via oral consent forms from key informants, healthcare workers, patients, clients, and community members prior to participation in any part of the study. The sample size and distribution of each survey, KII, and FGD are presented in Table 1.

**Table 1:** Sample size by type of survey and region.

Study type	SNNPR	Oromia	Amhara	Tigray	Total
**Patient exit interview**	58	58	58	58	232
**HEW survey**	15	15	15	15	60
**Health Professional survey**	16	16	16	16	64
**KII**	2	2	2	2	12
**FGD**	1	1	1	1	4

Secondary data sources, Table 2, included cost, administrative, and implementation data from Ministry of Health (MoH) reports which listed the number of health facilities, health posts, personnel, services provided, salaries, as well as HEP recurrent and capital costs between 2005 to 2017. Secondary financial and administrative data were collected from reports gathered from the MoH and its subsidiaries at all levels of the system (zonal, regional, and national). Population data and other regional demographic information were collected from the MoH and other government sources. All data were fully anonymized at the time of access as they were collapsed to the level of the facility, administrative district, or state.

**Table 2:** Breakdown of the tools and data utilized for the analyses.

Analysis	Surveys and Tools Utilized	Data Utilized
**Cost Analysis**	Primary and Secondary Data Collection from all surveys	Personnel, recurrent and Capital Costs
**Equity and Empowerment Benefit**	Patient Exit Interviews	Utilization and cost at health post compared to health center and hospital
		Income, education, geographic and empowerment classifications
**Employment Benefit**	FGD with Community Members	Local Multiplier Effect
**Health/Productivity Benefit**	LiST Tool	Lives Saved and Productivity Analysis with GDP per capita and Value of a Statistical Life

### Lives saved

Utilizing MoH documents and other literature [5, 22–25], main HEW responsibilities were categorized into 28 separate activities: 23 clinical, and five non-clinical. Using responses from the HEW and health professional surveys, as well as the number of patients seen by HEWs and other health professionals at HCs and HPs, a score was calculated for each activity estimating how much a HEW was contributing to each activity in comparison to all other health worker cadres. For example, a contribution of 100% indicated a HEW was entirely responsible for a given task and that all the lives saved for said task could be attributed to the HEP.

The Lives Saved Tool (LiST) was then used to map all 28 clinical and non-clinical activities onto maternal and child lives saved using the standard methodology available in the LiST [22, 26]. The effectiveness and affected fractions embedded within LiST were used to estimate the number of lives saved from implementing a given level of service from coverage indicators in each of the four regions. The models embedded within LiST were used to estimate the reduction in mortality and lives saved for specific causes of death due to specified interventions mapped onto each of the clinical and non-clinical activities. Lives saved estimates were based on several data sources including Demographic and Health Surveys, Multiple Indicator Cluster Surveys (MICS), WHO, UNICEF, Countdown to 2030, and the Maternal and Child Survival Program (MCSP).

### Benefits analysis

Benefits were calculated for four domains: equity, empowerment, employment, and productivity. The equity and employment benefits were calculated using a similar methodology. Equity and empowerment benefits were valued as the savings in cost from improved access to HEWs at HPs in comparison to the additional cost of visiting a HC or hospital for women of different equity (income, education, and geography) and empowerment categories. All respondents were asked to estimate the total number of visits and out-of-pocket costs for visits made to their local HP in the last year, as well as for a proxy counterfactual of the number of times a year they would have gone to a HC or hospital if there was no HEW in the local community. A difference-in-difference method was then used to estimate changes in health service utilization and costs between these two scenarios in each equity and empowerment group. Additionally, an empowerment index was calculated to categorize each female respondent into the following two categories: lower (below the median), and higher (above the median). These groups were based on a standard set of empowerment questions from the Demographic and Health Surveys and related literature [27–31]. In order to account for the double-counting of women who were in similar categories of income, education, and geography, only the marginal return was utilized above the category with the smallest return on investment. For example, if the valued returns were $100 for education, $300 for income and $400 for geography, the marginal return would include the $100 for education, the marginal return for income ($300-$100 = $200) and geography ($400-$300 = $100) for a total return of $400. Using results from the difference-in-difference models, the visits saved for each woman were estimated as an additional percentage of yearly visits saved to all HCs in each region. These were then multiplied by the average out-of-pocket expenditure and travel costs incurred for each equity category as well.

To estimate employment benefits, a local economic multiplier approach was utilized. This multiplier considered the percent of HEWs salary reported being spent in their local areas, defined as within 50 km of the health post. This value was then multiplied by data gathered on the local spending patterns of several shopkeepers from each region, who reported on the percent of their revenue spent locally on supplies, rent, taxes, etc., within 50km. These local multiplier numbers were calculated for each region and subsequently applied to all HEW salaries.

To estimate the final benefit component of the HEP, the productivity gain, the following two approaches were used: the standard method, and the Value of a Statistical Life (VSL) approach. The first approach was calculated as the number of lives saved and deaths averted due to HEW services multiplied by per capita gross domestic product (GDP) of the country [17, 20, 32]. For this standard method, we assumed any ‘saved lives’ would enter the workforce at age 18 and exit at 56 years old. We also considered a GDP growth rate of 2.5% and a discount rate of 5% for this calculation as well. In the VSL procedure, lives were considered to have additional worth beyond pure economic value. This approach takes into consideration an individual’s own valuation of the benefits of saving a life [33]. Since this approach depends on willingness to pay, we estimated a ratio multiplier for Ethiopia using information from other studies. The ratio multiplier was estimated at 3.0 and was used to estimate the upper bound of productivity [34].

### Cost analysis

Costs associated with the HEP included personnel, recurrent, and capital measures. Using the methodology presented in the Second Panel on Cost-Effectiveness in Health and Medicine [35, 36], our ROI included the following costs: formal healthcare sector costs (cost of providing services, as well as out-of-pocket costs for the patient), informal healthcare sector costs (transportation, and patient time costs), as well as broader, non-healthcare sector social costs/benefits, such as productivity, employment, equity, and empowerment. Given the lack of formal reimbursement processes for healthcare services in Ethiopia, an activity-based costing method, consisting of personnel, recurrent and capital items, was used to estimate the cost of providing services related to the HEP. Personnel cost included salaries, benefits, and bonuses received by HEWs, health professionals, and other staff working under the HEP at the HC and the HP levels. However, administrative and program management costs incurred by administrative districts and national staff were excluded. For each level, only those personnel who were directly working during the start-up of the HEP were included. If a category of personnel worked less than full time on the HEP, their level of effort was estimated accordingly. Recurrent costs included HEW recruitment and training, as well as essential health package kits, equipment, uniforms, utilities, printing, and training activities. Capital costs included HP construction, HP equipment, and office supplies. Capital goods included all equipment, buildings, and other capital items (motorbikes and vehicles) provided during the program. All item quantities were based on either the number provided to each respective HP, HC, or administrative office. All capital items were amortized using standard amortization rates which considered total costs, a three percent depreciation rate, and estimated useful life. ‘Useful life’ refers to the average number of years a respective item lasts before it needs to be replaced.

All cost data were collected for the current year (2018) as well as the start-up year (2005). As stated above, all initial costs were also deflated to 2005 using the World Bank Inflation Calculator. Additionally, all costs were converted from Ethiopian Birr to USD based on the 2018 exchange rate (1 ETB = 0.036 USD). Using 2018 cost data, per HEW costs were adjusted for inflation (15.6 percent annually) to calculate per HEW implementation costs for 2008 to 2017 as well as the total costs for all HEWs over the 10-year HEP.

### Return on investment

Return on investment was calculated at the total benefits divided by the total costs plus investments over the period 2008 to 2017. Total benefits included equity, empowerment, employment, and productivity benefits. Total costs included personnel, recurrent, and capital costs for the investment year (2005) as well as for implementation years between 2008–2017. ROI was calculated separately for all four regions (Tigray, SNNPR, Oromia, and Amhara).

### Estimation of the lives saved by the HEP

We estimated the number of maternal and child lives saved using the LiST [37, 38]. This enabled us to estimate the contribution each specific health service made towards saving lives. However, since there were multiple facilities and community-based health service providers working in tandem, we had to single out the contribution of each HEW to distinguish between the providers. HEWs stationed at HPs, as well as Health Cadres stationed at affiliated HCs were asked to report the number of services delivered listed in Table 5. The HEW contribution was then estimated by dividing the number of services delivered by HEWs by the total number of services delivered for each HC/HP network.

This study received ethical approval from the Institutional Review Boards at Brandeis University (Protocol #18146) and the Ethiopian Ministry of Health, who reviewed, approved, and accepted all the materials submitted to the Brandeis IRB as sufficient and issued letters of support to conduct field work in the four study regions. The Ethiopian Ministry of Health had jurisdiction to provide IRB oversight. Additional information regarding the ethical, cultural, and scientific considerations specific to inclusivity in global research is included in the Supporting Information (S1 Checklist).

## Results

### Lives saved

Table 3 shows the number of maternal and child lives saved for each clinical and non-clinical activity ordered by decreasing HEW contribution. On average, 384 maternal lives are lost, and 51,084 child lives are saved from clinical activities in which HEWs contribute on average 48%, totaling 50,700 lives. Most of the activities the HEWs perform are geared towards caring for children. For example, for many of the clinical activities beneficial to mothers, such as providing tetanus toxoid vaccination and contraceptive use, HEWs have less than a 56% contribution. For some of the activities where the most children’s lives are saved, HEWs have a larger contribution (Vitamin A supplementation, measles-single dose, and PCV-three doses).

**Table 3:** HEW contribution (%) to clinical and non-clinical activities and maternal and child lives saved.

Clinical Activities	HEW Contribution	Maternal Lives Saved	Still birth and child lives saved
Vitamin A Supplementation	79	-	(4,520)
Measles-Single Dose	71	-	20,620
Rotavirus-Two Doses	71	-	4,139
Treatment for Moderate/Acute Malnutrition	71	-	4,221
Pneumococcal (PCV)-Three Doses	66	-	17,209
Vitamin A for Treatment of Measles	63	-	(13,216)
Tetanus Toxoid Vaccination	56	(17)	(634)
Zinc for Treatment of Diarrhea	45	-	4,638
Contraceptive Use	45	(693)	-
ORS for Childhood Diarrhea	43	-	3,205
Malaria Treatment	41	131	-
Oral Antibiotics for Pneumonia	39	-	3,837
Iron/Folate Supplementation in Pregnancy	39	-	204
Immediate Assessment and Stimulation	17	-	959
Antibiotics for Dysentery	13	-	(34)
Syphilis Detection and Treatment	2	-	51
Labor and Delivery Management	1	8	399
Neonatal Resuscitation	1	0	54
***Total [Mean]***	763 [42]	(570) [(143)]	41,132 [2,571]
**Non-Clinical Activities**			
Promotion of personal hygiene & waste disposal	71	-	3,316
Promotion of breast-feeding practices	59	-	874
Promotion of infant & young child feeding	58	-	1,745
Promotion of clean postnatal practices	54	186	4,017
***Total [Mean]***	242 [61]	187 [187]	9,953 [2,488]
***Grand Total [Mean]***	1005 [48]	(384) [(77)]	51,084 [2,554]

*Note: Negative values (lives lost) are denoted in parentheses.

Table 4 details the number of lives saved between 2008–2017 in Amhara, Oromia, SNNPR, and Tigray. Due to the efforts of 37,949 HEWs, 50,700 total lives are saved, with SNNPR capturing the highest number out of the four regions (16,835). While Tigray shows the smallest number of total lives saved (6,081), it is the only region where maternal lives saved could be attributed to the HEP. In Amhara, Oromia, and SNNPR, there are female lives that could have been saved but due to reductions in coverage of certain indicators over time these lives are lost.

**Table 4:** Lives saved (2008–2017).

Region	Children	Women	Total Lives Saved	No. of HEWs
Amhara	15,375	(94)	15,281	9,849
Oromia	12,696	(193)	12,503	16,561
SNNPR	16,951	(116)	16,835	9,286
Tigray	6,062	19	6,081	2,253
**4 Regions**	**51,084**	**(384)**	**50,700**	**37,949**

*Note: Negative values (lives lost) are denoted in parentheses.

Table 5 describes the value of each of the benefits from 2008–2017. The largest benefits are the productivity gains, which are $3,818 per HEW, per year. Since the productivity benefit is calculated based on the estimated lives saved and mortalities averted, this implies the economic benefit is the largest when more lives are saved.

**Table 5:** Value of benefits by type from the Health Extension Program (2008–2017).

**Equity Benefit**	$932/HEW/year
Low Income vs. high income	$113,064,103
Rural vs. urban	$106,775,056
No education vs. any level of education	$133,741,005
Total amount	$350,580,164
**Empowerment Benefit**	$467/HEW/year
Low empowerment vs. high empowerment	$177,359,537
**Employment Benefit**	$532/HEW/year
Total Employment Benefit	$201,908,648
**Productivity Benefit**	
Economic Value	$3,818/HEW/year
Total Economic Value	$1,448,735,423
Value of a Statistical Life (VSL)	$11,453/HEW/year
Total Value of a Statistical Life (VSL)	$4,346,206,270
**Total Benefits (2008–2017)**	
Economic Value	$2,181,583,722
Value of a Statistical Life (VSL)	$5,079,054,619
**Average Benefits per HEW per Year**	
Economic Value	$5,749
Value of a Statistical Life (VSL)	$13,384

The equity benefit is the second largest at $932 per HEW, per year. Utilizing the lower level HEW services at HPs instead of traveling to distant, higher-level hospitals reduces out-of-pocket expenses on commuting for health care services. Since the HEP’s inception, HEWs are most beneficial for low income, less educated, and rural women, who save $113 million, $133 million, and $107 million, respectively, by using HEW services within their own communities; taking into consideration double counting. Improved equity returns more than $350 million back by enabling women to stay in their community and receive medical care.

Finally, the employment benefit and empowerment benefits, while the least remunerative, still accrue $532 per HEW, per year, and $467 per HEW, per year, respectively.

### Costs

The costs of the implementation of the HEP over the period 2008–2017 are in Table 6. Total costs include personnel, recurrent, and capital costs. From 2008–2017, Ethiopia’s government invested $1,368,537,668 into the HEP, 46% of which were recurrent/other operating costs. Costs vary greatly depending on the region, with the highest expenditures in Oromia ($611,528594) and lowest in Tigray ($84,849,343).

**Table 6:** The final costs of the HEP by region USD.

Cost Category	Amhara 2008–2017	Oromia 2008–2017	SNNPR 2008–2017	Tigray 2008–2017	Total 2008–2017
Personnel	97,458,318	219,705,005	94,289,209	31,462,202	442,914,735
Other Operating	170,930,957	269,991,957	159,904,810	34,228,398	635,056,123
Capital	76,290,556	121,831,632	73,285,880	19,158,743	290,566,810
Total	344,679,832	611,528,594	327,479,899	84,849,343	1,368,537,668

### Return on investment

As illustrated in Table 7, when using the VSL procedure, the ROI was $3.71 for the four regions included in the study; every $1 invested in HEW yielded a net benefit of approximately $3.71. Using this method, the ROI sees the most benefit in the regions of Tigray ($6.64) and SNNPR ($4.65). In contrast, the simple method procedure yields an ROI of $1.59 for the four regions, which ranges from $1.27 to $2.57 in Oromia and Tigray, respectively.

**Table 7:** Financial return on investment—facility costs only (2008–2017), revised numbers.

Region	Total Economic Benefits	Total VSL Benefits	Total Costs	ROI (Economic Evaluation)	ROI (VSL Evaluation)
Amhara	$628,464,902	$1,501,119,621	$ 344,679,832	**$1.82**	**$4.36**
Oromia	$775,526,166	$1,492,894,965	$ 611,528,594	**$1.27**	**$2.44**
SNNPR	$559,795,883	$1,521,583,592	$ 327,479,899	**$1.71**	**$4.65**
Tigray	$217,796,821	$563,456,441	$ 84,849,343	**$2.57**	**$6.64**
**Total**	$2,181,583,772	$5,079,054,619	$ 1,368,537,668	**$1.59**	**$3.71**

## Discussion

Ethiopia’s large investments into the HEP show promising results for future human resources for health programs. The results show positive returns for the HEP in all regions, with ROIs ranging from $1.27 on a $1 investment in Oromia, to $6.64 on a $1 investment in Tigray. Oromia, which saw the lowest ROIs, had sufficient health systems resources, suggesting that underutilization of these resources contributed to their relatively weak performance.

The results provide a robust examination of the return on investment, showing that the HEP has made important strides on improving health for women and children in Ethiopia. Given the great contributions HEWs made towards activities that directly saved the lives of children, it is not surprising that most of the positive impacts were in the number of children’s lives saved. In contrast, higher-level workers were completing activities identified as more effective at saving maternal lives, such as eclampsia and pre-eclampsia management, safe deliveries, and clean birth practices. Currently, HEWs have saved the most women’s lives through promotion of clean postnatal practices, though their capacity should be explored to determine whether they can take on more mother-oriented activities.

The results from this ROI are comparable to other ROIs. While other systems have not employed health extensions workers, other ROIs exist examining the impact of community health workers. While these types of cadres (CHWs and HEWs) have different roles, financing, and payment mechanisms, the return on such investments can be compared [39]. An analysis of an investment in CHWs across sub-Saharan Africa has estimated an ROI of 10:1, where every $1 invested in CHWs could yield a net benefit of $10. Since many of these community health workers are not paid, the costs of these programs are lower, increasing the ROI. However, many of the benefits overlap with the benefits estimated for the HEP in Ethiopia: increased productivity from a healthier population (number of lives saved using LiST, and potential economic impact of each life saved), avoidance of the high costs of health crises, and economic impacts of increased employment (with the spending multiplier for government spending programs in developing countries as 0.7) [20].

CHWs in the United States share some similarities with HEWs in that they originate from the community, have a deep understanding of local health issues, and are often paid or reimbursed for their work. Similarly to our results for Ethiopia, ROIs for CHWs in the US also vary significantly depending on the context and methodology [40–43]. A study from Nevada found an ROI of $1.81 for a pilot CHW program analyzing the impact of CHWs on increasing primary care visits, reducing acute visits, and affecting prescription costs [41]. In Kentucky, an ROI of $11.34 was determined for CHWs connecting inhabitants of the Appalachian region to medical, environmental, and social services [42]. In Colorado, the impact of CHWs on service utilization, charges, and reimbursements for a specific population was found to have an ROI of $2.28 [40]. Research from Connecticut reviewed how CHWs impacted diabetes control amongst Latinos, asthma control amongst children, and cardiovascular disease prevention complications, ultimately finding an ROI of $1.12, $1.86, and $2, respectively [43].

Another important aspect of the HEP is the impact the program had as part of an intentional system redesign. When the HEP was implemented in 2003, Ethiopia had only 0.03 physicians and 0.2 nurses per 1,000 population [44]. However, increasing the number of health care workers to achieve the WHO recommended density of 2.3 per 1,000 population was not financially feasible for countries like Ethiopia [45]. Therefore, the HEP was one piece of a larger system redesign undertaken by the government of Ethiopia to improve health care services for individuals, especially women and children, in Ethiopia. This analysis captures the impact of this aspect of their system redesign.

There were several limitations to this study. One limitation of the analysis that needs further investigation is the impact of the HEP on quality of care. As has been shown by other research, quality of care is lacking in many low-income countries, including Ethiopia [46, 47]. While our analysis focused on access to every health care service listed in Table 3, further research is warranted to understand if the HEP improved quality of care for women and children. Secondly, while HEWs mostly support maternal and child health, they also provide basic treatment services for all populations as defined in their minimum service package [48]. Such activities are not accounted for in the lives saved calculations because the necessary intervention and mortality data for these interventions were not available, which LiST requires for the lives saved calculations. Therefore, lives saved may be slightly underestimated given that maternal and child health are the priority for HEWs. In addition, some values of the calculated benefits were based on self-reported out-of-pocket costs for health care services, which may have lower reliability or be somewhat underestimated. Similarly, while every attempt was made to use official MoH data to inform HEW enrollment numbers, salaries, costs, and levels of effort, some of these data were supplied by key informants. Finally, despite taking regional and costing variations into account within the analysis, there are additional sensitivity analyses across key benefits (equity and empowerment) that could be considered in future analyses.

## Conclusion

These results show that the HEP has important impacts on women and child health in Ethiopia. This is the first study to estimate a return on investment for such a large human resource investment, including not only productivity and employment, but also equity and empowerment. The results have been important for the further development of the HEP, especially in rural areas. The results of the study show that the HEP in Ethiopia is an example for other African nations of a program with strong economic benefits and long-term sustainability through formal integration as part of the government health system.

## Supporting information

S1 Checklist Inclusivity in global research. (DOCX)

S1 Data (XLSX)
