# Supplementary material for: Return on investments in the Health Extension Program in Ethiopia
Source: PLoS One. 2023 Nov 27;18(11):e0291958. doi: 10.1371/journal.pone.0291958 (PMC10681216; doi:10.1371/journal.pone.0291958)
Supplement: S1 Checklist — (DOCX) [file pone.0291958.s001.docx]

Inclusivity in global research

PLOS’ policy on inclusivity in global research aims to improve transparency in the reporting of research performed outside of researchers’ own country or community and ensures that PLOS publications reporting global research adhere to high standards for research ethics and authorship. Authors of relevant research articles may be asked to complete the questionnaire below, which outlines ethical, cultural, and scientific considerations specific to inclusivity in global research. This questionnaire may be requested when researchers have travelled to a different country to conduct research, if research uses samples collected in another country, research with Indigenous populations or their lands, or if research is on cultural artefacts. Researchers travelling to another country solely to use laboratory equipment will not normally be required to complete the questionnaire. However, the questionnaire can be requested at the journal’s discretion for any submission – if you have been requested to complete this questionnaire by the PLOS journal you submitted to, please do so.

Please complete the questionnaire below and include this as a Supporting Information file with your manuscript. Note that if your paper is accepted for publication, this checklist will be published with your article in the supporting information files. Please ensure that you reference the checklist in the main body of your manuscript. We suggest adding a subsection ‘Inclusivity in global research’ to your Methods section and adding the following sentence: “Additional information regarding the ethical, cultural, and scientific considerations specific to inclusivity in global research is included in the Supporting Information (SX Checklist)”

The questions have been designed to be applicable to a wide range of study types, and there are subsections for both human subjects research and non-human subjects research. If any of the questions are not relevant to your research please mark them as “N/A” as appropriate.

**Ethical considerations, permits and authorship**

*This section is applicable to all research types.*

Provide details as to who granted permissions and/or consent for the study to take place in the Methods section of your manuscript. This should include the names of **all** ethics boards, governmental organizations, community leaders or other bodies that provided approval for the study. If individuals provided approval refer to these people by their role or title but do not list their name(s).

Reported on page number: 14

If there were any deviations from the study protocol after approval was obtained please provide details of these changes in the Methods section of your manuscript.
Did this study involve local collaborators that are residents of the country where the research was conducted or members of the community studied? If you do not have any authors from said communities, please provide an explanation for this below.

Reported on page number: N/A

Yes, local collaborators, at the national and local levels, were involved in the study. As detailed in the revised Figure 1, “Stakeholder involvement using KII and Focus Groups with administrative and clinical personnel in the health care system, community members, including patients, and local business owners.” In addition, one of the key authors of the study is an Ethiopian citizen (Leulsegged Kasa). USAID and the Ethiopian MOH reviewed the protocol and draft report.

Everyone listed as an author should meet PLOS’ criteria for authorship and all individuals who meet these criteria should be included in the author byline, rather than the acknowledgements. For further information please see the journal’s Authorship Policy.

**Human subjects research (e.g. health research, medical research, cross-cultural psychology)**

Did you obtain written informed consent from a representative of the local community or region before the research took place? How did you establish who speaks for the community? Details of written informed consent obtained from study participants should be reported separately in the Methods section of your manuscript.

As detailed in the methods section, informed consent was collected through oral consent forms from key informants and healthcare workers, patients, clients, and community members prior to participation in the survey or key informant interviews.

How did members of the local community provide input on the aims of the research investigation, its methodology, and its anticipated outcome(s)?

Local community members were involved in the study through key informant interviews and focus group discussions. The community members included in the study were administrative and clinical personnel in the health care system, community members, including patients, and local business owners but did not provide input on the aims of the research. USAID and the Ethiopian MOH reviewed the protocol and draft report, providing comments on the draft report.

When engaging with the local community, how did you ensure that the informed consent documents and other materials could be understood by local stakeholders?

The informed consent documents and other materials (surveys) were translated orally in the local language and delivered orally by bilingual interviewers.

Will the findings of the research be made available in an understandable format to stakeholders in the community where the study was conducted (e.g. via a presentation, summary report, copies of publications, etc.)? Please provide details of how this will be achieved.

The project has been completed and some preliminary findings were shared with local stakeholders, including USAID and Ethiopian MOH. The final publication will be shared once it is available.
